# Nijmegen breakage syndrome: 25-year experience of diagnosis and treatment in Ukraine

**DOI:** 10.3389/fimmu.2024.1428724

**Published:** 2024-06-28

**Authors:** Oksana Boyarchuk, Larysa Kostyuchenko, Hayane Akopyan, Anastasiia Bondarenko, Alla Volokha, Anna Hilfanova, Ihor Savchak, Liliia Nazarenko, Nataliia Yarema, Olha Urbas, Iryna Hrabovska, Oleksandr Lysytsia, Andrii Budzyn, Oksana Tykholaz, Mariana Ivanchuk, Olha Bastanohova, Erika Patskun, Nataliia Vasylenko, Yuriy Stepanovskyy, Liudmyla Chernyshova, Halyna Makukh

**Affiliations:** ^1^ Department of Children’s Diseases and Pediatric Surgery, I.Horbachevsky Ternopil National Medical University, Ternopil, Ukraine; ^2^ Clinic of Pediatric Immunology and Rheumatology, Western Ukrainian Specialized Children’s Medical Centre, Lviv, Ukraine; ^3^ Department of Diagnostic of Hereditary Pathology, Institute of Hereditary Pathology of the National Academy of Medical Sciences of Ukraine, Lviv, Ukraine; ^4^ Department of Pediatrics, Immunology, Infectious and Rare Diseases, European Medical School, International European University, Kyiv, Ukraine; ^5^ Department of Pediatrics, Pediatric Infectious Diseases, Immunology and Allergology, Shupyk National Healthcare University of Ukraine, Kyiv, Ukraine; ^6^ Department of Pediatrics, Cherkasy Regional Children’s Hospital, Cherkasy, Ukraine; ^7^ Department of Pediatrics, Ivano-Frankivsk National Medical University, Ivano-Frankivsk, Ukraine; ^8^ Department of Pediatrics Oncohematology, Volyn Regional Territorial Mother and Child Health Care Center, Lutsk, Ukraine; ^9^ Department of Bone Marrow Transplantation and Intensive Megadose Chemotherapy and Immunotherapy, National Specialized Children’s Hospital “OHMATDYT”, Kyiv, Ukraine; ^10^ Department of Propedeutics of Pediatric Diseases with Patient Care, National Pirogov Memorial Medical University, Vinnytsya, Ukraine; ^11^ Center of Specialized Pediatric Care, Poltava Regional Clinical Hospital named after M. V. Sklifosovsky, Poltava, Ukraine; ^12^ Department of Faculty Therapy, Uzhhorod National University, Uzhhorod, Ukraine; ^13^ Outpatient Department, Kherson Regional Children’s Hospital, Kherson, Ukraine; ^14^ Department of the Research and Biotechnology, Scientific Medical Genetic Center LeoGENE, Lviv, Ukraine

**Keywords:** Nijmegen breakage syndrome, *NBN* gene, c.657_661del5 variant, malignancies, diagnosis, incidence, clinical analysis, immunological characterization

## Abstract

**Introduction:**

Nijmegen breakage syndrome (NBS) is an autosomal recessive disorder, characterized by microcephaly, immunodeficiency, and impaired DNA repair. NBS is most prevalent among Slavic populations, including Ukraine. Our study aimed to comprehensively assess the prevalence, diagnosis, clinical data, immunological parameters, and treatment of NBS patients in Ukraine.

**Methods:**

We conducted a retrospective review that included 84 NBS patients from different regions of Ukraine who were diagnosed in 1999-2023. Data from the Ukrainian Registry of NBS and information from treating physicians, obtained using a developed questionnaire, were utilized for analysis.

**Results:**

Among 84 NBS patients, 55 (65.5%) were alive, 25 (29.8%) deceased, and 4 were lost to follow-up. The median age of patients was 11 years, ranging from 1 to 34 years. Most patients originate from western regions of Ukraine (57.8%), although in recent years, there has been an increase in diagnoses from central and southeastern regions, expanding our knowledge of NBS prevalence. The number of diagnosed patients per year averaged 3.4 and increased from 2.7 to 4.8 in recent years. The median age of NBS diagnosis was 4.0 years (range 0.1-16) in 1999-2007 and decreased to 2.7 in the past 6 years. Delayed physical development was observed in the majority of children up to the age of ten years. All children experienced infections, and 41.3% of them had recurrent infections. Severe infections were the cause of death in 12%. The second most common clinical manifestation of NBS was malignancies (37.5%), with the prevalence of lymphomas (63.3%). Malignancies have been the most common cause of death in NBS patients (72% of cases). Decreased levels of CD4+ and CD19+ were observed in 89.6%, followed by a reduction of CD3+ (81.8%) and CD8+ (62.5%). The level of NK cells was elevated at 62.5%. IgG concentration was decreased in 72.9%, and IgA - in 56.3%. Immunoglobulin replacement therapy was administered to 58.7% of patients. Regular immunoglobulin replacement therapy has helped reduce the frequency and severity of severe respiratory tract infections.

**Conclusion:**

Improvements in diagnosis, including prenatal screening, newborn screening, monitoring, and expanding treatment options, will lead to better outcomes for NBS patients.

## Introduction

Nijmegen breakage syndrome (NBS) is an autosomal recessive disorder classified among inborn errors of immunity (IEI), characterized by microcephaly, immunodeficiency, impaired DNA repair, leading to high sensitivity to ionizing radiation and an increased risk of malignancy.

Chromosomal instability syndrome was first highlighted in the article by Hustinx et al. (1), where a patient with microcephaly, growth delay, selective IgA deficiency, and chromosomal rearrangements was reported. The etymology of the disorder’s name is associated with the city of Nijmegen, where the first two cases of NBS were described in 1981 (2).

NBS is most prevalent in Eastern European populations, particularly in Poland, the Czech Republic, and Ukraine, where the frequency of homozygous carriage of the “Slavic” pathogenic variant (c.657_661del5) of *NBN* gene approaches 1:155 (3). The prevalence of NBS in Ukraine is 1.3 per 1,000,000 population, with the highest frequency of cases in western regions (up to 20 cases per 1,000,000 individuals) (4). NBS is also common in isolated Slavic groups in southeastern Germany, where the carrier frequency is 1:34 (5). In the United States, carriers of the pathogenic variant c.657_661del5 are usually patients of Eastern European descent (6).

Malignancy is a manifestation of NBS and is sometimes the presenting symptom in heterozygous carriers of the pathogenic variant of the *NBN* gene (2). *NBN* gene in NBS encodes the protein Nibrin, which is part of the NBN/Mre11/Rad50 complex and, interacting with ATM kinase, halts the cell cycle with DNA repair. Double-strand DNA breaks may occur during normal replication processes and as a result of the impact of damaging agents on DNA (7).

In patients with NBS, inefficient repair of DNA breaks occurs in the absence of Nibrin, disrupting V(D)J recombination processes during lymphocyte differentiation (8). Impaired lymphocyte proliferation and differentiation lead to a decrease in their number, the formation of antigen-recognition domains of immunoglobulins, and the T-cell receptor (9).

*NBN* plays a crucial role in neurogenesis (9, 10). DNA damage affects the development of neurons in various parts of the brain. Microcephaly can be caused by alterations in neural progenitor cells in response to DNA damage. Impaired cell proliferation and differentiation, and the absence of Nibrin in NBS patients result in growth retardation (11).

Chromosomal instability has been demonstrated in the karyotype of patients with NBS in several clinical studies (1, 12–14), most often revealing inversions and translocations involving two different loci on chromosomes 7 and 14 (14, 15). Breakpoints are located in the chromosomal bands 7p13, 7q35, 14q11, and 14q32, regions of T-cell receptor genes and the human immunoglobulin heavy chain gene. Cytogenetic studies in NBS patients’ lymphocytes reveal fusion of the ends of adjacent chromosomes, indicating telomere dysfunction (16, 17). Since telomere shortening triggers cell aging, the role of Nibrin in telomere maintenance likely also contributes to growth retardation in NBS patients.

Thus, studies have shown the widespread prevalence of NBS among Slavic populations and identified the role of the Nibrin protein in the clinical manifestations of the disease. The wide prevalence of NBS poses important tasks for doctors and scientists in Ukraine: establishing molecular-genetic diagnostics of the syndrome, adequate clinical management of patients to prevent oncological and other complications of congenital combined immunodeficiency, and increasing attention to NBS among children with microcephaly.

Previous studies examined the course of NBS in a cohort of 26 patients from 1999-2007 (18). In addition, 14 patients from the Lviv region included in the ESID registry were included in the study by Wolska-Kuśnierz et al. in 2015 (19), and 56 patients from Ukraine were included in the cohort of Eastern Slavic population NBS patients, which studied the prevalence of malignancies (4). Hence, there have been no comprehensive investigations conducted in recent years on the prevalence, clinical presentations, and complications of NBS within the Ukrainian population. Our study aimed to comprehensively assess the prevalence, diagnosis, clinical data, immunological parameters, and treatment of NBS patients with 657del5 variant in *NBN* gene in Ukraine.

## Materials and methods

### Patients

We conducted a retrospective study that included 84 NBS patients from different regions of Ukraine. In 82 patients, the diagnosis was suspected based on clinical data, and in two patients - based on positive neonatal screening results. The diagnosis was confirmed by genetic testing in all patients. In 75 cases, the diagnosis was confirmed at the Institute of Hereditary Pathology in Lviv, Ukraine; in four cases - at the Scientific Medical Genetic Center LeoGENE, Ukraine; in three cases - at the Invite laboratory, USA; in one case - at the genetic laboratory of the National Specialized Children’s Hospital “OHMATDYT”, Kyiv; and in one case - at a clinic in Italy, where hematopoietic stem cell transplantation (HSCT) was performed. A homozygous c.657_661del5 variant in *NBN* gene was detected in 83 patients, and heterozygous c.657_661del5 and c.995-2A>G variants were found in one patient.

The research adhered to the guidelines outlined in the 1975 Declaration of Helsinki (revised in 2000) and received approval from the Ethics Committee of I. Horbachevsky Ternopil National Medical University (Minutes № 75). All participants and/or their parents provided written informed consent.

To analyze demographic data and clinical signs, we used data from the Ukrainian Registry of IEI (20). We also surveyed immunologists with experience observing patients with NBS, using a detailed questionnaire developed by Professor Larysa Kostyuchenko. This questionnaire included questions about various aspects of the disease: clinical features, laboratory indicators, and treatment methods.

Evaluating previous studies on NBS in Ukraine, we divided our analysis into three periods: 1999-2007, 2008-2017, and 2018-2023. This approach allowed us to reconcile data and identify certain patterns in the disease dynamics.

Additionally, to prevent new cases of NBS in high-risk families, a prenatal diagnostics program has been developed at the Institute of Hereditary Pathology. The program has already been successfully implemented in five families of heterozygous carriers of c.657_661del5 variant in the NBN gene.

### Genetic diagnosis

Molecular-genetic analysis of the pathogenic variant c.657_661del5 of the *NBN* gene was conducted in probands with clinical suspicion and phenotypic features of NBS. DNA isolation and purification were performed using the phenol extraction method and salting-out method. For postnatal diagnosis, venous blood samples collected in EDTA tubes or dried blood spots (DBS) were used. In cases of prenatal diagnosis, DNA was isolated from chorionic villi or amniotic fluid. *In vitro* DNA sequence amplification was performed using the polymerase chain reaction (PCR) method with the following primer sequences 5’-CAGATAGTCACTCCGTTTACAA-3’ and 5’-TTACCTGTTTGGCATTCAAA-3’ and DreamTaq PCR MM (Thermo Scientific). DNA fragment separation was carried out by DNA electrophoresis in a 10% polyacrylamide gel prepared in Tris-borate buffer. Electropherograms were scanned on an ultraviolet transilluminator. The obtained signals were compared with length markers, and based on this, the sizes of the obtained fragments were detected. In the presence of the pathogenic variant c.657_661del5 of the *NBN* gene in a homozygous state, the size of the PCR fragment was 176 bp, compared to the normal allele - 181 bp. Sanger Sequencing was performed using the QuantumDye sequencing kit (Quantum Seq) on SeqStudio Capillary electrophoresis (Applied Biosystems).

### Statistical analysis

Statistical analysis was performed using STATISTICA 10. We used descriptive statistics. Qualitative variables are shown as absolute frequencies and percentages. Quantitative variables were tested using the Kolmogorov**–**Smirnov test or histogram for normal distribution and are expressed as median and interquartile range (IQR), when appropriate. For categorical data, chi-squared tests were used. Values of *p*<0.05 were considered significant.

## Results

### Characteristics of NBS patients

Among 84 NBS patients, there were four pairs of siblings. Forty-four were males (52.4%). The median age of all patients was 11 years, ranging from 1 to 34 years. Overall, 61.9% of patients were alive at the time of data collection, with 28 (33.3%) deceased. Four patients were lost to follow-up for various reasons. The median age of live patients was 12 years, ranging from 1 to 34 years. Twelve living patients are currently over 18 years old (21.4%), and four patients are over 30 years old. The median age of deceased patients was 9 years, ranging from 4 to 16 years.

**Figure 1:**
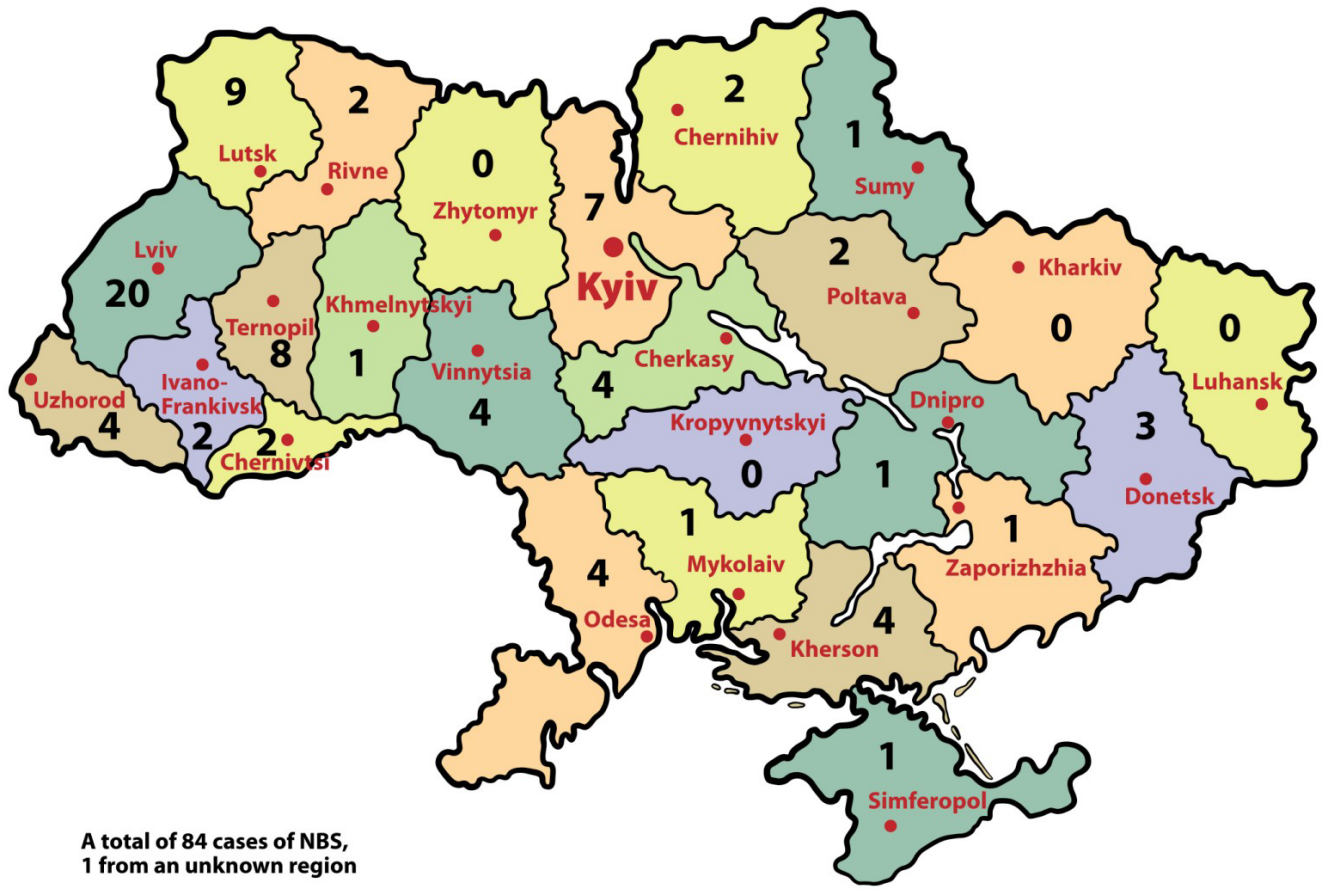
Geographical distribution of NBS patients (n) by regions of Ukraine.

The distribution of patients according to the regions of Ukraine is shown in **Figure 1**.

**Figure 2:**
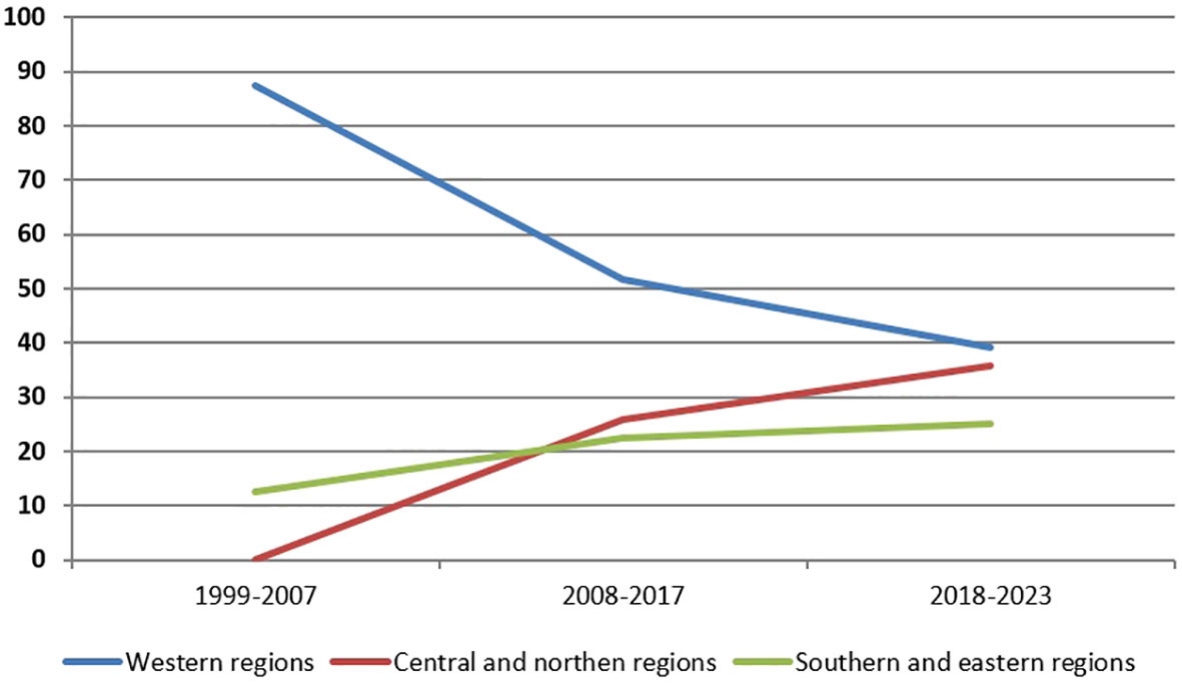
Trends in diagnosing NBS patients (%) depending on the period (years) and region.

Analysis of diagnosed patients depending on the region of Ukraine was conducted. Overall, the largest proportion of patients (57.8%) is registered in the western regions, but in recent years, there has been a trend of increasing the number of patients in the central and northern regions: from 0% in the period 1999-2007 to 35.7% in the last 6 years (**Figure 2**).

The number of diagnosed patients per year averaged 3.4 and increased from 2.7 to 4.8 in recent years (**Table 1**). The median age of NBS diagnosis from 1999 to 2023 was 4.0 years (range 0.1-16). It decreased from 4.0 years in 1999-2007 and 5.5 years in 2008-2017 to 2.7 years in 2018-2023. The median age of diagnosis also varied depending on the region: in western regions, it was 2.6 years (range 0.1-16 years), in central and northern regions – 5.5 years (range 0.1-13 years), and in southern and eastern regions – 6 years (range 0.4-16 years); p=0.0838 between western and other regions.

**Table 1:** Dynamics of the number of born and diagnosed NBS patients and median age of diagnosis.

Criterion	1999-2007	2008-2017	2018-2023	Total
Number of diagnosed patients, n	24	31	29	84
Number of diagnosed patients per year	2.7	3.1	4.8	3.4
Age at diagnosis, years; Me (range)	4.0 (0.1–16)	5.5 (0.1-16)	2.7 (0.1-13)	4.0 (0.1–16)
Number of born patients	28	31	12	71*
Number of newborns, thousands	3,737	4,686	1,618	10,041
Incidence in newborns	1: 133,000	1: 151,000	1: 135,000	1:141,000

*13 NBS patients were born in 1989-1998 years.

### Prenatal diagnosis and medical-genetic counseling

Prenatal diagnosis were carried out in 5 families of heterozygous carriers of pathogenic variant c.657_661del5 of *NBN* gene. In the family where two children with NBS died from complications of Hodgkin’s lymphoma, 4 prenatal diagnostic procedures were conducted. In 2 cases, a homozygous pathogenic variant in the *NBN* gene was identified, while in the other two cases, a heterozygous variant and normal *NBN* genotype were identified, and healthy children were born. In the remaining four families at high risk of NBS, prenatal diagnosis was performed only once, during the subsequent pregnancy after the birth of a child with NBS. A homozygous pathogenic variant of *NBN* was identified in 3 of the investigated cases, and only one healthy child was born in one family.

### Clinical manifestations

Data on the clinical course of NBS were provided for 58 patients (69.0%). Microcephaly was noted in all, 4 (6.9%) children were born prematurely, but in 41 (70.7%) newborns, the birth weight was less than 3000 g, while the body length was less than 50 cm in 25 (43.1%) children. The median birth weight was 2800 g, ranging from 1800 to 3800 g (**Table 2**). The median body length at birth was 50 cm, ranging from 44 to 54 cm. At birth, only one patient had a head circumference of 34 cm, which corresponds to the lower limit of the norm, while in the rest, it ranged from 27 to 33 cm, with a median of 30 cm. Subsequently, microcephaly was observed in all children, with a median head circumference deviation of -6.5z at 1 year, ranging from -4.3 to -8.1z, and a median of -6.1z at 5 years, ranging from -3.8 to -7.5z.

**Table 2:** Physical development of NBS patients depending on age.

Age, years	Me	IQR	Range (min; max)	Less than -2z	n
Weight, z-score					
At birth	2800 g	2580; 3050 g	1800; 3800 g		58
1	-1.97	-2.25; -0.77	-5.03; 2.0	19; 55.9%	34
3	-2.30	-4.56; -1.16	-8.03; 2.71`	13; 59.1%	22
5	-2.07	-3.73; 0.72	-7.87; 3.83	16; 64%	25
10	-1.93	-2.93; 0.18	-4.0; 2.28	10; 56.3%	16
14	0.19	-0.39; 1.18	-4.28; 1.18	3; 33.3%	9
18	0.29		-0.57; 0.1		2
Length/height, z-score					
At birth	50 cm	48; 51 cm	44; 54 cm		58
1	-1.17	-2.69; -0.11	-4.05; 0.77	13; 41.9%	31
3	-0.47	-1.6; 1.02	-5.88; 4.90	6; 28.6%	21
5	-0.71	-2.60; 0.67	-7.64; 0.89	8; 33.3	24
10	-1.80	-2.50; 1.17	-4.20; -0.84	7; 50%	14
14	-0.91	-1.44; 0.10	-2.18; 0.10	1; 12.5%	8
18	-0.31		-3.7; 0.31		2

Delayed physical development was observed in the majority of children up to the age of 10 (**Table 3**). Deviations in weight were more frequent and pronounced than in body length/height. After 10 years, a positive trend was observed in both weight and height.

**Table 3:** Frequency of clinical manifestations in NBS patients (n=58).

Sign	n	%
Craniofacial abnormalities	58	100
microcephaly	58	100
characteristic faces	55	94.8
sloping forehead	56	96.6
prominent and/or hooked nose	39	67.2
small chin	54	93.1
upslanting palpebral fissures	30	51.7
abnormal ears	46	79.3
Skin manifestations		
café au lait spots	27	46.6
nevi	4	6.9
vitiligo	14	24.1
Infections		
Sporadic respiratory tract infections	34	58.6
Recurrent infections	24	41.3
bronchitis	16	27.6
otitis	13	22.4
sinusitis	10	17.2
pharyngitis/tonsillitis	4	6.9
chronic bronchitis/bronchoectasis	4	6.9
stomatitis	8	13.8
chronic conjunctivitis	3	5.2
urinary tract infections	5	8.6
*Herpes simplex* infection	8	13.8
Clinically relevant EBV infection	2	3.4
Pneumonia	13	22.4
Enterocolitis	6	10.3
Pyodermia	3	5.2
Mycoses	4	6.9
Warts	1	1.7
*Molluscum* *contagiosum* infection	2	3.4
Tuberculosis	1	1.7
Sepsis	2	3.4
HCV infection	2	3.4
HBV infection	2	3.4
COVID-19	2	3.4
Autoimmune diseases	9	15.5
neutropenia	4	6.9
psoriasis	1	1.7
arthritis	4	6.9
Malignancies	35/80	43.8
lymphoma	22	27.5
leukemia	8	10.0
rabdomyosarcoma	1	1.3
ovarian cancer	1	1.3
undefined	3	3.8
Allergies	5	8.6

Dysmorphic facial features, such as characteristic faces, sloping forehead, prominent and/or hooked nose, small chin, upslanting palpebral fissures, and abnormal ears, were observed with varying frequency (**Table 3**). The predominant skin manifestations included café au lait spots (46.6%) and vitiligo (24.1%).

All children experienced infections; however, in 34 (58.6%) cases, they were sporadic during the observation period, did not require intensive medication intervention, and manifested as common infections. In 24 (41.3%) cases, the infections were recurrent. Among them, respiratory tract infections were most common: bronchitis (27.6%) and sinusitis (17.2%). Pneumonia was reported in 22.4% of children. Recurrent otitis media (22.4%), stomatitis (13.8%), and urinary tract infections (UTIs) (8.6%) were frequently reported by physicians. Herpes simplex infections, mainly labialis, were observed in 13.8% of children. Four children had hepatitis (hepatitis B and hepatitis C in two children each), two had sepsis unrelated to immunosuppression in HSCT, and one child had tuberculosis.

Information about COVID-19 was available for three patients. In children receiving immunoglobulin replacement therapy, the disease had a mild course. In an adult patient (34 years old), COVID-19 manifested with high fever and respiratory symptoms but did not require hospitalization. After SARS-CoV-2 infection, this patient developed arthritis of the knee joint.

Severe infections were the cause of death in three children (12%).

The second most common clinical manifestation of NBS was malignancies, which occurred in 35 out of 80 patients (43.8%) at the time of examination. Of the 35 patients with malignancies, 24 were boys (68.6%) and 11 were girls (31.4%), p=0.0399.

Among malignancies, lymphomas were most commonly observed (62.9%), followed by leukemias (22.9%), and in isolated cases, other types of malignancies were identified (rhabdomyosarcoma, ovarian cancer). In three cases, the diagnosis was unclear. Of the 22 patients with lymphomas, 18 had non-Hodgkin lymphomas (NHL) and 4 had Hodgkin lymphoma. Among the NHL, diffuse large B-cell lymphoma (DLBCL) was observed in 10 patients, other types of B-cell lymphoma – in 3, T-cell lymphoblastic lymphoma (T-LBL) – in 4, and peripheral T-cell lymphoma (PTCL) – in one patient. The median age of children at the time of malignancy diagnosis was 9.5 years, ranging from 3 to 15 years. Currently, 22 patients (62.9%) with malignancy have passed away, while the rest are in remission or continue treatment.

Autoimmune and allergic manifestations in NBS patients were less common, observed in 15.5% and 8.6% respectively (**Table 4**). Among autoimmune phenomena, arthritis (6.9%), neutropenia (6.9%), and psoriasis (1.7%) were noted. Allergies included atopic dermatitis (4; 6,9%), allergic rhinitis (2; 3.4%), and bronchial asthma in 1 child.

**Table 4:** Immunoglobulins and lymphocyte subpopulations in NBS patients (n=48).

Parameter	Deviation	n (%)	Me (interquartile range, IQR)	Range (min-max)
Lymphocyte, cells/mm^3^	decreased	31 (64.6)	1580 (803; 2310)	38-10100
CD3+, cells/mm^3^	decreased	32 (81.8)	847 (510; 985)	190-2510
CD4+, cells/mm^3^	decreased	43 (89.6)	407 (295; 475)	60-2050
CD8+, cells/mm^3^	decreased	30 (62.5)	284 (116; 491)	96-1072
CD19+, cells/mm^3^	decreased	43 (89.6)	106 (78; 180)	10-875
NK, cells/mm^3^	increased	30 (62.5)	393 (283; 570)	2-1360
	decreased	2 (4.2)		
Ig G, g/l	decreased	35 (72.9)	3.70 (2.08; 8.01)	0.80 – 14.7
Ig A, g/l	decreased	27 (56.3)	0.13 (0.02; 0.50)	0 – 2.23
Ig M, g/l	increased	10 (20.8)	0.75 (0.41; 1.1)	0 – 2.20
	decreased	4 (8.3)		
Ig E, IU/ml	decreased	18/30 (60)	12 (1; 104)	0.1-1317
	increased	4/30 (13.3)		

### Immunological examination

Immunological data were provided for 48 (57.1%) NBS patients. Decreased lymphocyte levels were observed in 64.8% of them (**Table 4**). The most common reductions were in absolute values of CD4+ and CD19+ (89.6%), followed by CD3+ (81.8%) and CD8+ (62.5%). Meanwhile, the level of NK cells was more frequently elevated (62.5%). The most common reduction among immunoglobulins was in IgG concentration (72.9%), followed by IgA (56.3%). The median value of IgG was also low, at 3.7 g/l. Immunoglobulin M was within the normal range in most cases, but in 20.8% it was elevated, and only in 8.3% it was decreased. In 60% of children with measured IgE, it was decreased, but in 4/30 (13.3%) it was elevated, and in 2 patients it exceeded 1000 IU/ml.

### Treatment

There is currently no specific treatment for NBS. The main methods of treatment for NBS patients include immunoglobulin replacement therapy, prophylactic antibiotic therapy, and HSCT. HSCT has shown clinical benefits for immunodeficiency and hematologic cancer in patients with NBS (21). However, the high risks associated with this treatment are linked to increased radiosensitivity and impaired tolerance to chemotherapy. There is also a risk of secondary malignancies (21). Additionally, ethical considerations must be taken into account, especially when neurological impairments progress, as congenital chromosomal fragility and radiosensitivity, will remain unchanged even with a successful HSCT. Therefore, the decision to pursue transplantation should be considered individually for each patient, taking into account the clinical severity of immune defects and other manifestations.

Immunoglobulin replacement therapy was administered to 37/63 (58.7%) NBS patients with low IgG levels and/or recurrent and severe infections. Patients received regular immunoglobulin at a 400-800 mg/kg dose once every 28 days. Regular prophylactic immunoglobulin replacement therapy became available in Ukraine only in 2006. Until then, immunoglobulin replacement therapy was episodic. The median age of starting immunoglobulin therapy was 3.5 years, ranging from 3 months to 16 years. Prophylactic antibiotic therapy was administered in 13/63 (20.6%) patients with recurrent respiratory symptoms. Other treatment options included antiviral and antifungal therapy when necessary.

HSCT was performed in 4 patients, three of whom died. HSCT was performed in Italy, Poland, Turkey, and Ukraine. In three patients, it was performed in remission of malignancies, while in one child, considering the complicated infectious history, progressive decline in T- and B-lymphocytes.

Unfortunately, the first experience of HSCT in Ukraine in a 3-year-old NBS patient was unsuccessful. The patient did not have malignancy but had a severe recurrent infection and progressive T-cell lymphopenia. A compatible related donor was not available. With the help of the Ukrainian bone marrow donor registry, a compatible non-relative donor (9/10 match at the A locus) was found in the International bone marrow donor registry, as a 10/10 match donor was unavailable. After comprehensive pre-transplant evaluation, the patient underwent reduced-intensity conditioning (RIC) according to the EBMT/ESID Guidelines for HSCT for primary immunodeficiency (modified preparative regimen for patients with radiosensitivity). Engraftment was achieved within the expected timeframes: neutrophil on day +11, platelet on day +13, erythrocyte on day +15. Molecular genetic study of donor chimerism in peripheral blood by PCR on days +27, +57, +90, and +197 confirmed complete donor chimerism - 100%.

Starting from the early post-transplant period, the patient experienced immune complications: graft-versus-host disease (GVHD), which was resistant to glucocorticoid therapy. Therapy with mesenchymal stem cells, vedolizumab also did not have a positive effect. The patient developed gastrointestinal bleeding and multi-organ failure progressed. The child died on day 253 after transplantation.

## Discussion

The diagnosis of NBS in Ukraine began in 1998-2001 at the Department of Hematology of the Lviv Regional Children’s Specialized Hospital (now Western Ukrainian Specialized Children’s Medical Center) (22, 23). Molecular diagnosis for the first patients was conducted at the Institute of Medical and Human Genetics Charité, Berlin, Germany (3, 7, 24). The molecular-genetic diagnosis of NBS in Ukraine began in 2004 at the Institute of Hereditary Pathology, Lviv.

Professor Larisa Kostyuchenko played a significant role in the study, diagnosis, and treatment of NBS patients in Ukraine (18, 23, 25). The first cohort of patients (1999-2007) were described by Dr. Kostyuchenko in 2009 (18).

The prevalence of NBS patients with the Slavic variant in the western regions of Ukraine as of 2017 was 3.6 per 1,000,000, reaching up to 24 per 1,000,000 in some areas of this region (4), one of the highest in Europe and the world. The highest prevalence of NBS was reported in Poland and the Czech Republic, where it was 3.1 per 1,000,000 (19, 24).

According to the results of genetic testing of the 657del5 variant of the *NBN* gene in randomly selected Gutherie newborn screening cards of healthy newborns, the heterozygous carrier frequency in the Czech Republic was 1:130, in Poland 1:253, and in the Lviv population of Ukraine 1:182 (3). Consequently, the expected frequency of NBS in the Lviv region was 1:133,000 newborns. However, when calculating based on the frequency of verified NBS cases from 2001-2010, it turned out to be significantly higher, at 1:34,000, with heterozygous carriers frequency at 1:95 newborns (20). These results align with the rates observed in the northern and eastern regions of Wielkopolska province, Poland (1:76-77) (26) and the vicinity of Novi Sonch, which is geographically close to Lviv (1:90) (3).

However, according to our data, the overall frequency of NBS in Ukraine is 1:141,000 newborns. Overall, our study showed a trend towards improving the diagnosis of NBS over the past 25 years. The increase in the number of diagnosed patients and the shift in the percentage of patients from the western regions of Ukraine to other regions demonstrates improved awareness of NBS among physicians, better accessibility to genetic diagnostics, and population migration. The war may have affected access to diagnostics in eastern and southern regions, which are frontline areas (27), although there is a steady trend towards improved diagnosis in central regions.

The median age of diagnosis has become younger - from 5.5 in 2008-2017 to 2.7 years in 2018-2023. It is worth noting that in 2022, expanded neonatal screening for 21 diseases, including severe combined immunodeficiency (SCID), was introduced in Ukraine. A pilot project of newborn screening using TREC and KREC assay to determine T- and B-lymphopenia confirmed the diagnostic capabilities for NBS (28). It was found 100% sensitivity for TREC copies and 56% sensitivity for KREC copies for the detection of NBS (29). In 2023, NBS was diagnosed in two children due to positive results of newborn screening for SCID confirmed by genetic analysis.

Wolska-Kuśnierz et al. (19) 2015 described 149 NBS patients with the Slavic variant of *NBN* from the European Registry for PID, among which the majority were patients from Poland (118) and Ukraine (14). Overall, the median age of NBS patients in our cohort is slightly lower than in the mentioned study (11 versus 14.3 years). The percentage of deceased patients in our study is slightly lower (33.3% versus 39%), but the children died at a younger age (median age of deceased 9 years versus 11.1 years).

Overall, 6.9% of patients were born prematurely, but 70.7% had low birth weight (less than 3000g), while a body length of less than 50 cm was observed in 43.1% of children. Our results coincide with the data described in previous studies (6, 19). Although there were no premature births in the previous cohort, 42.9% of mothers experienced spontaneous abortions during previous pregnancies (18).

Data reflecting the dynamics of physical development in NBS are limited. We have shown that up to the age of 10, most patients lag in weight, and a significant portion (from 28.6% to 50%) in body length/height. Another study also showed significantly lower anthropometric indicators in NBS patients than in the healthy population (11). As in our observations, Varon et al. (6) also note that growth deficiency improves with age. The improvement in the physical condition of children with NBS may be associated with better diagnosis as well as improvements in socio-economic status, treatment options for NBS, and associated conditions that arise with it. Microcephaly was observed in all patients with a median deviation in head circumference of -6.5z at 1 year.

Our data related to infections differ slightly in some indicators from the results described by Wolska-Kuśnierz et al. in 2015 (19). Their study observed recurrent infections without severe course or complications in 23% of patients, but 43% experienced severe respiratory problems. According to our study, a lower frequency of pneumonia (22.4% versus 46.4%), chronic bronchitis, and bronchiectasis (6.9% versus 23.6%) was also noted, but recurrent sinusitis (17.2% versus 8.2%) and recurrent labial herpes infection (13.8% versus 0.9%) were more common. Data on SARS-CoV-2 infection in three patients showed a mild course, which in one patient was complicated by arthritis three weeks after COVID-19 (29, 30). The decrease in the frequency of recurrent respiratory infections in recent years may be associated with both earlier NBS diagnosis and better access to immunoglobulin replacement therapy, as well as patient isolation during the COVID-19 pandemic. It should be noted that infections were the cause of death in three cases (12% of deaths), which coincides with the results of previous studies, where 14% of patients died from pulmonary infections with respiratory insufficiency (19).

Malignancies were diagnosed in 43.8% of children, which aligns with the results of other studies where oncopathology was diagnosed in 42% (19) and 45% (4) of NBS patients. Malignancies were more common in boys (p=0.0399), which is consistent with the results of another study (4). Lymphomas predominated in patients with malignancies (62.9%), as reported in previous studies (4, 19, 22). Among lymphomas, aggressive DLBCL type prevalated (45.5%). The median age of children at the time of malignancy diagnosis was 9.5 years, which also corresponds to the results of other studies (19, 23).

Autoimmune and allergic manifestations are also characteristic of IEI (31, 32), although they were less common in NBS patients (15.5% and 8.6%, respectively), which aligns with reports from other researchers (19).

The analysis of immunological changes in the presented cohort of patients mostly corresponded to those changes described previously (19, 33). The number of NK cells was increased in the majority of NBS patients. Another study indicated that in most patients, the number of NK cells was within the normal range (79%), with elevated levels in 13% and decreased levels in 8% (19). However, the study of Gregorek et al. (33) confirmed our results, as the number of NK cells in NBS patients was significantly higher than in healthy controls (32.3% vs. 9.7%, p < 0.01) (33). Researchers also demonstrated significant deviations in the maturation process of peripheral T lymphocytes in NBS patients compared to healthy individuals. The substantial increase in populations of effector T cells in NBS patients may be associated with increased susceptibility to malignancies and a milder clinical course than expected, considering T-cell lymphopenia (34). Researchers did not find a correlation between T-cell lymphopenia and the frequency and severity of respiratory infections (19). It was also reported that abnormalities may progress over time (33); however, this hypothesis lacks credible evidence.

Changes in humoral immunity were most commonly reflected in decreased levels of IgG (72.9%) and IgA (56.3%), which also corresponds to the data from previous studies (62% and 57%, respectively) (19). The authors noted a correlation between the degree of IgG and IgA deficiency and the severity and/or chronicity of respiratory tract infections (19).

The treatment of NBS patients remains challenging due to the lack of standardized therapeutic options. In all cases, the degree of immunodeficiency should be assessed, and any infectious complications require prompt decision. Immunoglobulin replacement therapy allows control for the infectious syndrome in the majority of patients. These patients respond poorly to treatment of malignancy due to radiosensitivity and impaired tolerance to chemotherapy. Allo-HSCT may be considered as a treatment option aimed at correcting the immune system. However, it is still controversial and requires further research, for the optimal preparative regimen before HSCT, graft sources, choice of graft-versus-host disease prophylaxis method, peculiarities of pre- and post-transplantation support, and so on (21, 35).

Thus, the conducted study revealed the wide prevalence of NBS in Ukraine and a tendency towards improving its diagnosis. However, considering the high carrier frequencies of NBN in Czech, Ukrainian, and Polish populations (3), the number of patients in Ukraine should be higher, indicating a significant number of undiagnosed cases. Increasing awareness among the medical community and raising public awareness may improve the diagnosis of IEI, including NBS in Ukraine (36, 37). Implementing newborn screening for T- and B-cell lymphopenia may also help improve early diagnosis of NBS, as well as careful registration and vigilance regarding patients with microcephaly, growth delay, and malignancy. Prenatal diagnosis is important for families with cases of children’s death at an early age from severe or recurrent infections or malignancies, and the presence of individuals in the family with microcephaly. Frequent miscarriages should also raise suspicion of NBS. Considering the high frequency of the 657del5 variant of the *NBN* gene, predictive genetic testing may be considered. Early diagnosis will allow for appropriate prevention of infections, avoiding radiation exposure for diagnosing infections and other pathologies, and selecting the appropriate strategy for treating malignancies.

### Strengths and limitations of the study

A noteworthy aspect of this study is that it is the first comprehensive analysis of the prevalence, clinical, and immunological characteristics of patients from various regions of Ukraine over 25 years. Previous studies mainly focused on data from patients in western regions during the initial 10 years of diagnosis, or the prevalence of malignancies was limitedly studied. The cohort of Ukrainian patients with Slavic variant in the *NBN* gene is one of the largest in the world which enhances the generalizability of the findings. The analysis was conducted on the growth and weight parameters of children with NBS from birth to 18 years, which has been insufficiently addressed in previous publications.

However, a limitation of this study is its retrospective data collection, which posed challenges in obtaining certain data, as well as the possibility of missing data or inaccuracies in the medical records and conducting a thorough assessment of result completeness. Transitioning to adult care resulted in worsened patient monitoring, making it difficult to track data. Another limitation of this study is that we identified the c.657_661del5 variant of the *NBN* gene, while around 10% of patients with NBS harbor alternative pathogenic variants, which could influence the epidemiological results. The prevalence of NBS in the Ukrainian population may be higher with improved diagnosis and surveillance. Nonetheless, analyzing data longitudinally allows for identifying diagnostic trends and determining ways to improve both diagnosis and the management of NBS patients.

### Conclusion

The cohort of diagnosed NBS patients with the 657del5 variant in the *NBN* gene in Ukraine currently comprises 84 individuals, making it one of the largest in Europe and the world. Most patients originate from western regions of Ukraine (57.8%), although in recent years, there has been an increase in diagnoses from central and southeastern regions, expanding our knowledge of NBS prevalence. Malignancies have been the most common cause of death in NBS patients, accounting for 72% of cases. Regular prophylactic immunoglobulin replacement therapy has helped reduce the frequency and severity of severe respiratory tract infections.

Improvements in diagnosis, including prenatal screening, newborn screening, monitoring, and the expansion of treatment options, will lead to better outcomes for NBS patients.

## Data availability statement

The raw data supporting the conclusions of this article will be made available by the authors, without undue reservation.

## Ethics statement

The studies involving humans were approved by I.Horbachevsky Ternopil National Medical University. The studies were conducted in accordance with the local legislation and institutional requirements. Written informed consent for participation in this study was provided by the participants’ legal guardians/next of kin.

## Author contributions

OBo: Conceptualization, Data curation, Formal analysis, Project administration, Software, Supervision, Writing – original draft, Writing – review & editing. LK: Conceptualization, Data curation, Investigation, Writing – original draft, Writing – review & editing. HA: Visualization, Writing – original draft, Writing – review & editing. ABo: Data curation, Writing – original draft, Writing – review & editing. AV: Writing – original draft, Writing – review & editing, Data curation. AH: Data curation, Writing – original draft, Writing – review & editing. IS: Writing – original draft, Writing – review & editing. LN: Writing – original draft, Writing – review & editing. NY: Writing – original draft, Writing – review & editing. OU: Writing – original draft, Writing – review & editing. IH: Writing – original draft, Writing – review & editing. OL: Writing – original draft, Writing – review & editing. ABu: Writing – original draft, Writing – review & editing. OT: Writing – original draft, Writing – review & editing. MI: Writing – original draft, Writing – review & editing. OBa: Writing – original draft, Writing – review & editing. EP: Writing – original draft, Writing – review & editing. NV: Writing – original draft, Writing – review & editing. YS: Writing – original draft, Writing – review & editing. LC: Writing – original draft, Writing – review & editing. HM: Investigation, Methodology, Writing – original draft, Writing – review & editing.
